# Natural-Origin Bioadhesive Injectable Hydrogels Composed of Polyphenol and Chitosan with Antibacterial Activity for Wound Healing

**DOI:** 10.3390/gels12050448

**Published:** 2026-05-20

**Authors:** Hongyu Zheng, Shikui Wu, Yujie Liu, Yuzhu Zhang, Yushu Xing, Jianye Wang, Xin Yue, Lijun Sun, Xiao Li, Ying Zhang, Jiannan Ma, Xiaoli Du, Yan Xue, Juan Yu, Huiwen Zhang, Huanyun Wang

**Affiliations:** School of Pharmacy, Inner Mongolia Medical University, Hohhot 010110, China; zhenghongyuzz@163.com (H.Z.); wushikui@immu.edu.cn (S.W.); 2025110105@stu.immu.edu.cn (Y.L.); zhangyuzhu5114@gmail.com (Y.Z.); fdas.a@163.com (Y.X.); nmgwangjianye@163.com (J.W.); yinlucky@immu.edu.cn (X.Y.); iamsunlijun1984@126.com (L.S.); lx_leexiao@163.com (X.L.); 20070176@immu.edu.cn (Y.Z.); jiannanma@163.com (J.M.); 20120018@immu.edu.cn (X.D.); xyxy2172@163.com (Y.X.); yujuan4893@163.com (J.Y.)

**Keywords:** chitosan, hydrogel, wound healing

## Abstract

This study aimed to develop antibacterial polyphenol–chitosan hydrogel dressings and, more importantly, to compare how three structurally distinct low-cost natural polyphenols—protocatechuic acid (PCA), gallic acid (GA), and tannic acid (TA)—regulate hydrogel performance within the same chitosan platform. PCA, GA, and TA were incorporated into chitosan to obtain the corresponding hydrogels, denoted CS-PCA, CS-GA, and CS-TA. Scanning electron microscopy confirmed that all formulations possessed a three-dimensional porous network. Rheological characterization revealed favorable viscoelastic behavior for all polyphenol-containing hydrogels, with CS-TA showing the highest mechanical strength in the present system. The hydrogels also exhibited pH-responsive swelling, good tissue adhesion, self-healing ability, and injectability. In vitro antibacterial assays demonstrated activity against both Gram-positive and Gram-negative microorganisms, with CS-TA showing the most favorable overall antibacterial performance under the tested conditions. In a rat full-thickness wound model, hydrogel treatment accelerated wound closure, while H&E staining indicated enhanced granulation tissue formation, collagen deposition, and reduced inflammatory cell infiltration. Collectively, these findings support the use of polyphenol–chitosan composite hydrogels as promising wound-dressing candidates and highlight the value of a side-by-side comparison of PCA, GA, and TA for understanding structure–property–function relationships in this class of materials.

## 1. Introduction

The skin, which serves as the body’s primary physical barrier against the external environment, undergoes a highly coordinated series of biological processes during injury repair [1,2,3,4]. Wound healing typically progresses through three dynamic phases: inflammation, proliferation, and remodeling [5]. The inflammatory phase focuses on clearing pathogens and necrotic tissue [6]. The proliferation phase is characterized by granulation tissue formation, angiogenesis and re-epithelialization [7]. The remodeling phase culminates in repair through collagen reorganization and scar formation [8,9]. However, severe trauma or chronic wounds often disrupt this orderly sequence, leading to delayed healing, persistent infection, or even systemic complications [9].

Bacterial infection represents a major challenge in wound management. The growing problem of antibiotic resistance underscores the urgent need for novel antimicrobial materials. An ideal wound dressing should offer multiple functions: maintaining a moist environment, allowing gas exchange, effectively preventing infection, possessing suitable mechanical properties and adhesiveness, and having good biocompatibility and pro-healing capabilities [10,11]. Chitosan is widely used in wound-care materials because it is biocompatible, biodegradable, hemostatic, and intrinsically antibacterial. However, pristine chitosan hydrogels usually show limited mechanical strength and suboptimal wet-tissue adhesion [12,13]. Natural polyphenols offer a practical route to improve these weaknesses. Through hydrogen bonding, electrostatic interactions, π-related interactions, and, in some cases, covalent coupling, polyphenols can reinforce the chitosan network and simultaneously contribute antioxidant and antibacterial activity [14]. Representative examples have already been reported. Tannic acid-containing chitosan hydrogels have shown good adhesion, self-healing, and wound-repair capacity [15,16]. Gallic acid-modified chitosan systems have also been explored for wound management and tissue restoration [17]. In addition, a recent protocatechuic acid-grafted chitosan hydrogel promoted the repair of infected wounds [18].

At the same time, the current literature also shows two clear features. First, most studies focus on a single polyphenol and therefore do not allow direct comparison of how polyphenol structure changes hydrogel performance within the same chitosan framework [15,16,17,18]. Second, many recent high-performance systems achieve multifunctionality through more complex designs, for example, by combining chitosan with oxidized polysaccharides, metal ions, zinc oxide, carbon dots, or photothermal modules [19,20]. These strategies are effective, but they can also increase formulation complexity and make it harder to distinguish the specific role of the polyphenol itself. Therefore, although polyphenol–chitosan hydrogels are not new as a material family, a clear comparative analysis of structurally distinct, widely available polyphenols in one unified chitosan platform is still insufficient.

**Scheme 1:**
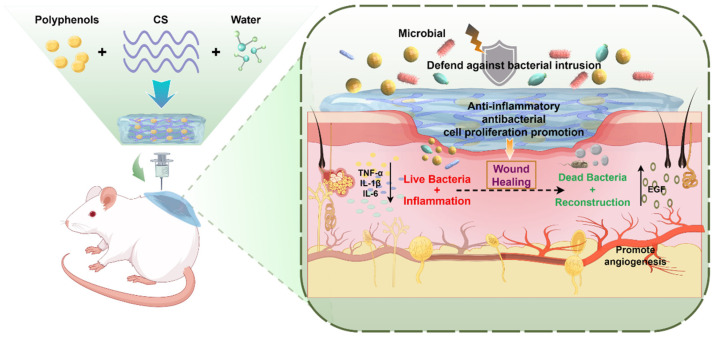
Synthesis routes of polyphenol–chitosan hydrogels and their mechanism of accelerating wound healing in vivo. In the figure on the right, the black arrow pointing upward indicates upregulation of cytokine levels, while the black arrow pointing downward indicates downregulation.

Based on this consideration, the present study selected three natural polyphenols with different molecular architectures, namely PCA, GA, and TA, and prepared the corresponding chitosan-based hydrogels under a comparable design framework. The purpose was not to claim the first preparation of a polyphenol–chitosan hydrogel, but to answer a more practical question: which type of low-cost natural polyphenol is more favorable for building a simple, bioadhesive, antibacterial chitosan hydrogel for wound healing? The novelty of this work therefore lies in the head-to-head comparison of PCA, GA, and TA within the same platform, the resulting structure–property–function analysis, and the identification of TA as the most promising candidate in this series. As a promising wound dressing, they are expected to restore the skin regeneration process and accelerate the healing of complex infected wounds (Scheme 1). The study provides not only a practical platform for developing natural-origin, multifunctional wound dressings but also molecular-level insights into the design of bioadhesive materials with tunable physicochemical and biological properties.

## 2. Results and Discussion

### 2.1. Synthesis and Characterization of the Hydrogels

**Figure 1:**
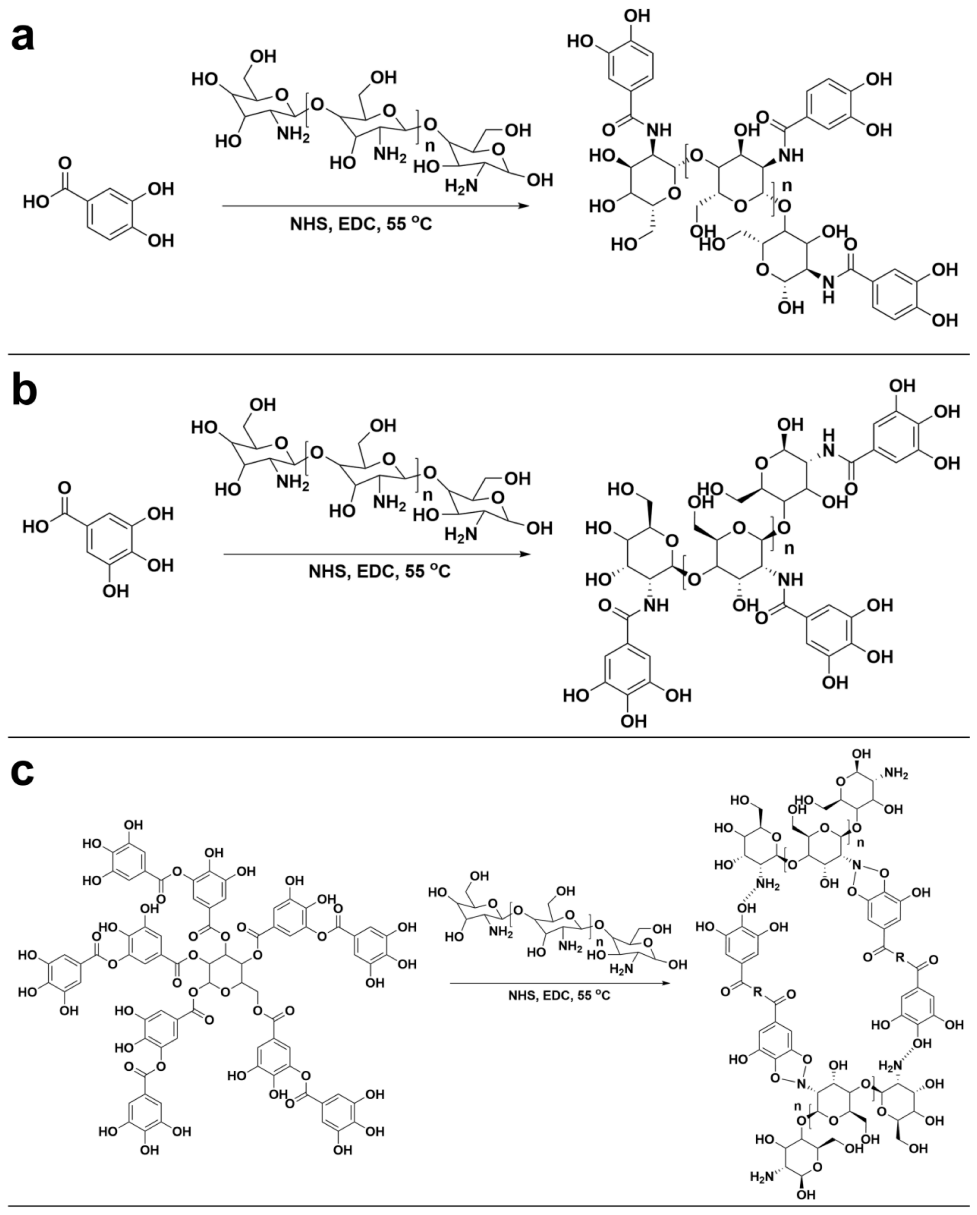
Reaction mechanisms of (**a**) CS-PCA, (**b**) CS-GA and (**c**) CS-TA.

Polyphenol-modified hydrogels with tunable mechanical properties, rapid shape adaptability, fast self-healing, and tissue adhesion were prepared by simple physical mixing. The hydrogels were prepared using a one-pot reaction method. Chitosan (CS), the polyphenol compound, and the crosslinker were sequentially added, yielding three polyphenol–chitosan hydrogels: CS-GA, CS-PCA, and CS-TA. With respect to GA and PCA, their active carboxyl groups were first activated by the carbodiimide agent (EDC) and N-hydroxysuccinimide (NHS), which were subsequently grafted onto the amino groups of chitosan via a Schiff base reaction to form the CS-GA and CS-PCA composite hydrogels, respectively. The reaction mechanism is illustrated in Figure 1a,b and is consistent with previous ones. As a macromolecular polyphenol, the interaction of TA and CS is more complex. CS was dissolved in dilute acid, and its amino groups were protonated to carry a positive charge (–NH_3_^+^). Moreover, TA dissolved in aqueous solution could be partially ionized through its phenolic hydroxyl groups, rendering it negatively charged while also acting as both a hydrogen bond donor and an acceptor. Hence, as shown in Figure 1c, when TA and CS are mixed, the galloyl groups in tannic acid can be activated by EDC/NHS to form amide bonds with the amino groups of chitosan. This covalent crosslinking, synergizing with multiple noncovalent interactions such as electrostatic attraction and hydrogen bonding, leads to the formation of a stable crosslinked product.

**Figure 2:**
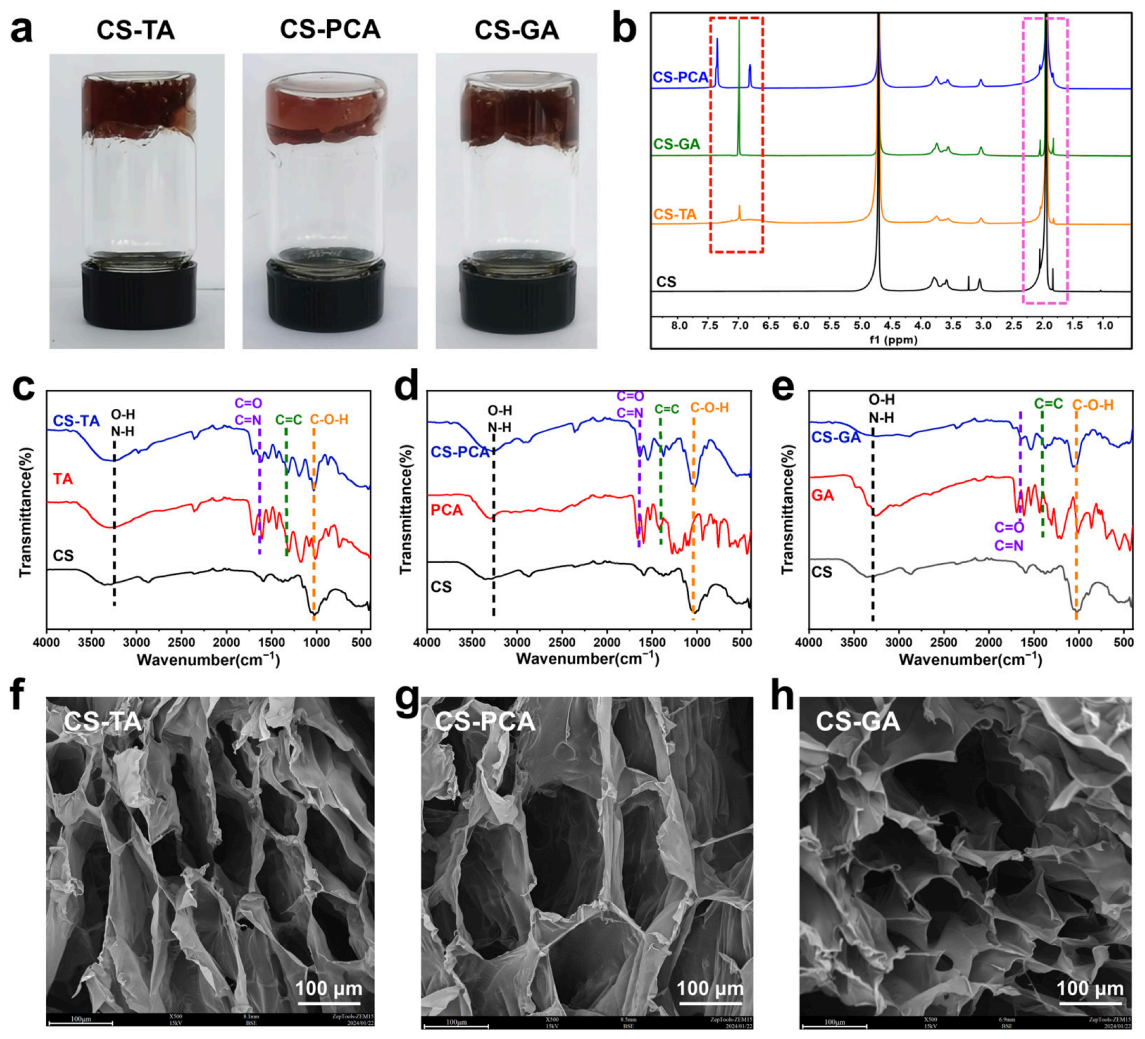
Physical properties and microstructures of the hydrogels. (**a**) Macroscopic appearances of CS-TA, CS-PCA, and CS-GA; (**b**) ^1^H NMR spectra (left red box: polyphenol peaks; right purple box: chitosan peaks); FT-IR spectra of the (**c**) CS-TA hydrogel, (**d**) CS-PCA hydrogel, (**e**) CS-GA hydrogel. SEM images of (**f**) CS-TA, (**g**) CS-PCA, and (**h**) CS-GA lyophilized hydrogels (SEM: ZEM15; 15 kV; BSE mode; ×500; scale bar = 100 μm).

To justify the selected formulation parameters, extensive single-factor preliminary experiments were first carried out, followed by a secondary screening using five candidate formulations with polyphenol solution volumes of 2, 4, 6, 8, and 10 mL at a fixed CS volume of 40 mL (Figure S1, Supporting Information). The resulting gel states differed visibly among formulations. Among them, the formulations containing 8 mL polyphenol solution showed better macroscopic homogeneity, gel integrity, and reduced flowability under vial inversion, and were therefore selected for the subsequent study. Based on these optimized conditions, CS-PCA, CS-GA, and CS-TA hydrogels were successfully prepared as homogeneous, non-flowing gels (Figure 2a).

The ^1^H NMR results further confirmed the successful introduction of polyphenol moieties into the CS system (Figure 2b). Compared with unmodified CS, CS-PCA, CS-GA, and CS-TA exhibited new aromatic proton signals in the δ 6.6–7.4 ppm region. These signals were consistent with the characteristic ^1^H NMR peaks of the corresponding pure compounds PCA, GA, and TA shown in the Supplementary Material (Figure S3, Supporting Information), and can therefore be assigned to the benzene–ring protons of the polyphenols. Meanwhile, the modified samples still retained the characteristic chitosan backbone signals, including the ring proton signals at δ 3.0–4.0 ppm and the residual acetyl methyl signal at δ 1.9–2.1 ppm, indicating that the main CS framework remained intact after modification. Together with the aromatic ring-related absorption observed in the UV–Vis spectra (Figure S2, Supporting Information), these results support the successful incorporation of PCA, GA, and TA into the chitosan system.

FT-IR analyses were performed on the CS, CS-PCA, CS-GA, and CS-TA hydrogels (Figure 2c–e), confirming the adsorption of PCA, GA, and TA onto the chitosan matrix, respectively. First, the infrared spectra of CS-TA, CS-PCA and CS-GA all retained the polysaccharide backbone structure of CS: ν _C-O-C_ (1100–1000 cm^−1^) and ν _C-OH_ (1150 cm^−1^), confirming the presence of CS in the polymers. Second, the characteristic aromatic ring peaks appeared in the spectra of the polymers: ν _C=C_ (1600 cm^−1^, 1450 cm^−1^), which preliminarily confirmed the successful grafting of polyphenolic compounds onto the CS backbone. Furthermore, compared with those of single CS and polyphenolic compounds, broader hydrogen bond absorption peaks ν _O-H_ and ν _N-H_ (redshift, 3600–3200 cm^−1^) were observed, further confirming the existence of hydrogen bonding interactions between the two components. More importantly, the emergence of the amide bonds ν _C=O_ (amide I, 1650 cm^−1^) and ν _C=N_ (redshift, amide II, 1550 cm^−1^) confirmed the occurrence of the Schiff base reaction. In addition, the FT-IR spectrum of CS-TA showed a stronger and broader absorption peak in the high wavenumber region (Figure 2c), which originated from the interaction between large numbers of phenolic hydroxyl groups on TA and CS.

Scanning electron microscopy (SEM) was used to characterize the microscopic morphologies of the CS-PCA, CS-GA, and CS-TA hydrogels (Figure 2f–h). All the hydrogel samples presented 3D networked porous structures, in contrast to the lamellar morphology observed in the pure CS hydrogel (Figure S4, Supporting Information). A gradual reduction in pore size was observed with increasing hydroxyl group content and molecular weight of the polyphenols, with CS-TA having the most compact porosity (Figure 2f). This trend can be attributed to the greater number of functional groups in TA, which provide more binding sites for interaction with CS and consequently lead to an increased crosslinking density. These findings suggest that the chemical structure, molecular weight, and interaction forces of the polyphenols play crucial roles in determining the final microstructure of the hydrogels.

### 2.2. Performance Evaluation of the Rheological Properties, Swelling Rate, Self-Healing, Degradation Behavior, Injectability, and Tissue Adhesion of the Hydrogels

**Figure 3:**
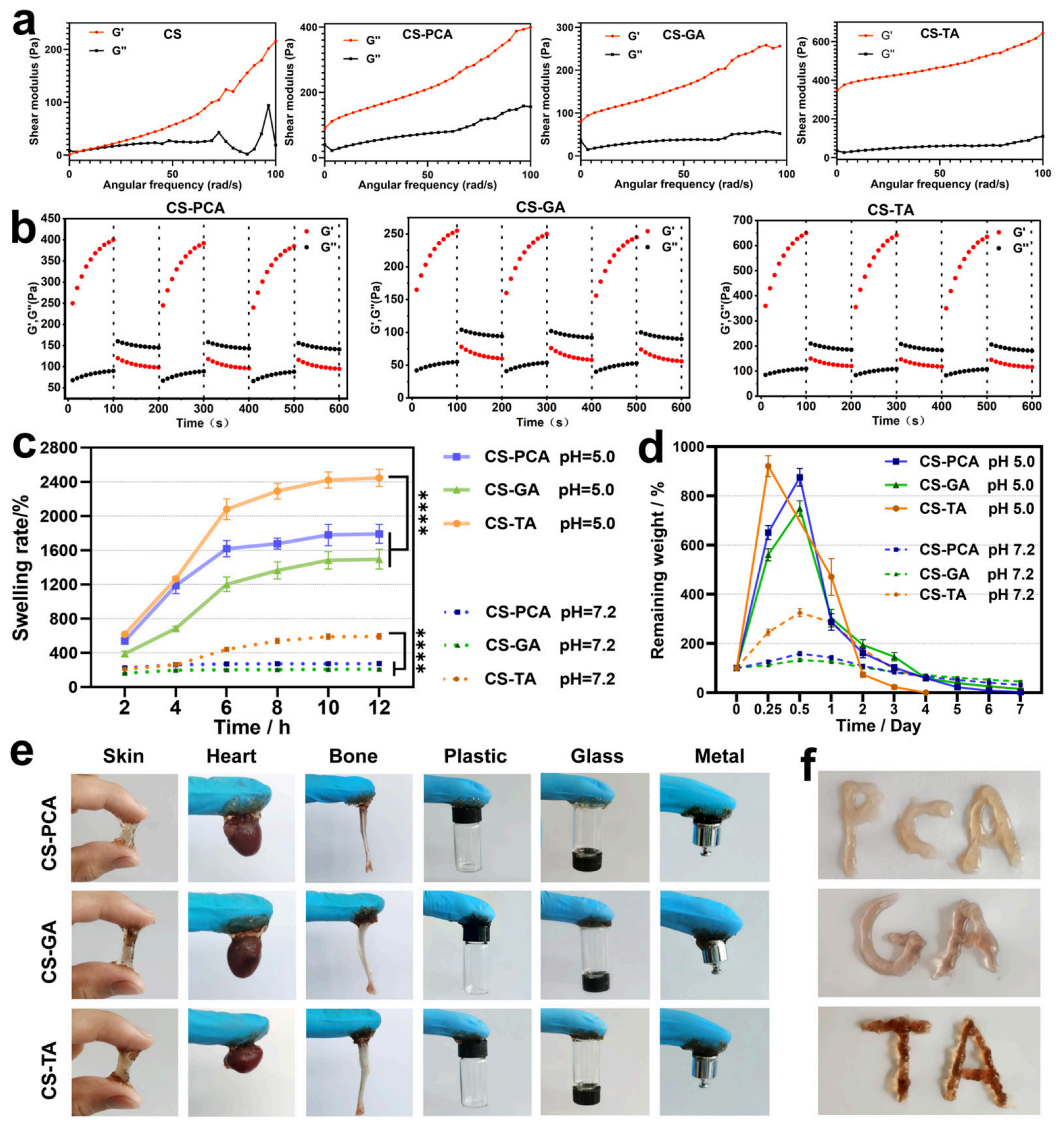
Characterization of the rheological properties, swelling behavior, degradation behavior, macroscopic adhesiveness, and injectability of the hydrogels. (**a**) Frequency sweep test of CS, CS-PCA, CS-GA, and CS-TA hydrogels, showing the storage modulus (G′) and loss modulus (G″) over an angular frequency range of 0.1–100 rad/s at a constant strain of 1%. (**b**) Step-strain rheological recovery behavior of CS-PCA, CS-GA, and CS-TA hydrogels under alternating strains of 5% and 100%, showing the reversible changes in G′ and G″ during repeated deformation–recovery cycles. (**c**) Swelling rate of CS-PCA, CS-GA and CS-TA in PBS (pH = 5.0/7.2) at 25 °C (*n* = 3). (**d**) Degradation behavior of CS-PCA, CS-GA, and CS-TA hydrogels in PBS at pH 5.0 and pH 7.2, expressed as remaining weight over time (37 °C, *n* = 3). (**e**) Adhesive properties of CS-TA, CS-PCA, and CS-GA to diverse material surfaces. (**f**) Demonstration of the injectability of CS-TA, CS-PCA, and CS-GA. Data are presented as mean ± SD. Statistical analysis was performed using two-way ANOVA followed by Tukey’s multiple-comparison test; **** *p* < 0.0001.

After a hydrogel is applied to a wound, especially at a joint, it should have good tissue adhesion and self-healing properties to address the shedding, damage, and deformation caused by external forces so that it will last a long time [21]. Therefore, we studied the mechanical behavior, self-healing, adhesive properties and swelling behavior of the hydrogels. The viscoelastic behaviors of chitosan (CS) hydrogel and its composites modified with various polyphenolic compounds (PCA, GA, TA) were systematically investigated through dynamic oscillatory strain sweep measurements. As illustrated in Figure 3a, distinct differences in the angular frequency (ω) dependence of the storage modulus (G′) and loss modulus (G″) were observed among the four hydrogels. The CS hydrogel exhibited structural weakness, as evidenced by its liquid-like behavior (G″ > G′) at low frequencies. This is characteristic of its weak physical network, which is susceptible to disruption under low shear conditions [22]. In contrast, the CS-PCA, CS-GA, and CS-TA composite hydrogels exhibited G′ > G″ throughout the entire tested frequency range (0.1–100 rad/s), demonstrating solid-like elastic behavior across all the measured conditions. These results confirm that all three phenolic cross-linkers successfully introduced stronger chemical and physical interactions, substantially enhancing the structural integrity and mechanical stability of the hydrogels. The higher G′ and G″ values observed for CS-TA serve as evidence for the formation of a denser and more stable three-dimensional network [23].

Swelling performance is not only an important indicator for evaluating the basic properties of hydrogel materials but also significant for their application prospects in the biomedical field. The swelling equilibrium curves of the polyphenol–chitosan composite hydrogels in PBS buffers at pH = 5.0 and pH = 7.2 indicated that within the first 6 h of the experiment, the mass of the hydrogels increased rapidly, with water molecules quickly penetrating the uniformly distributed pore structures of CS-GA, CS-PCA, and CS-TA (Figure 3c). The hydrogels reached swelling equilibrium at approximately 12 h. A comparison of the three different composite hydrogels revealed that the swelling rate of CS-TA was significantly greater than those of CS-GA and CS-PCA. This superior swelling performance of the CS-TA composite hydrogel can be attributed to the strong cross-linked structure formed between tannic acid molecules and chitosan, as well as the chemical characteristics of tannic acid itself. The high swelling properties of the CS-PCA, CS-GA, and CS-TA hydrogels are particularly important in biomedical applications such as skin repair and reconstruction, as sufficient swelling capacity ensures that the hydrogel continuously absorbs tissue exudate and maintains a suitable moist environment, thus promoting tissue regeneration and wound healing [24].

The remaining-weight behavior of hydrogels in aqueous media is an important parameter for evaluating their structural stability and potential residence behavior in wound-related environments [25]. In this study, the in vitro remaining-weight changes in CS-PCA, CS-GA, and CS-TA were examined in PBS at pH 5.0 and pH 7.2, which were used to simulate relatively acidic inflammatory conditions and near-neutral physiological conditions, respectively. As shown in Figure 3d. All three hydrogels exhibited mild and controllable pH-responsive degradation behavior. Among them, CS-GA showed the slowest degradation, making it suitable for chronic wounds requiring long-term protection; CS-TA degraded most rapidly, rendering it more appropriate for short-term applications or acute wounds; and CS-PCA displayed an intermediate profile, offering a balance between structural stability and degradability. These three hydrogels can be selectively applied according to different wound types and healing stages. Although the swelling, remaining-weight, rheological, injectability, and adhesion results provide preliminary pharmaceutical-relevant evidence for wound-dressing applications, release, permeation, diffusion, and accelerated stability tests were not performed in this study. The current stability evaluation was limited to remaining-weight changes in PBS at pH 5.0 and 7.2 for 7 days. Therefore, future studies should further assess polyphenol release/diffusion, wound-bed permeation, and stress stability under clinically relevant conditions.

The self-healing properties of the hydrogels were evaluated by both macroscopic observation and step-strain rheological tests. At the macroscopic level, the cut hydrogel pieces could rejoin spontaneously after contact (Figure S5, Supporting Information). Consistently, the step-strain recovery results showed that all hydrogels underwent a reversible transition between gel-like and disrupted states under alternating strains of 5% and 100% (Figure 3b). G′ was higher than G″ at 5% strain, whereas G′ decreased below G″ at 100% strain. After the strain was reduced to 5%, the moduli recovered rapidly over repeated cycles, confirming the self-healing capability of the hydrogels. CS-TA exhibited the highest modulus and the strongest recovery behavior among the three samples.

Injectability tests revealed that the CS-PCA, CS-GA, and CS-TA composite hydrogels also possessed good injectability (Figure 3f). The injectability of the hydrogel enhances material utilization efficiency by enabling precise delivery to the target site, thereby minimizing the waste of active components [26,27]. Furthermore, this property significantly broadens its potential clinical applications. A hydrogel with injectability and shape adaptability can fill various irregular wound shapes without external stimulation, making it an excellent candidate for wound dressings [28,29].

Polyphenol–chitosan composite hydrogels exhibit excellent compatibility and significant adhesion to various materials, making them biomaterials with broad application potential. This study revealed that these hydrogels can effectively adhere to various biological tissues, including skin, heart, bone tissue and plastic, glass, metal (Figure 3e). These moist surfaces simulate the real physiological environment in vivo, highlighting the great application prospects of polyphenol–chitosan composite hydrogels as wound dressings both in vitro and in vivo. This characteristic is particularly important because, in practical clinical applications, wound dressings need to maintain firm adhesion in moist or fluid-rich environments to provide continuous protection and treatment [30]. In addition to biological tissues, polyphenol–chitosan composite hydrogels can also adhere to various other materials, such as glass, plastic, and metal, without any surface pretreatment. This broad adhesion capability offers greater flexibility for hydrogel applications in different industrial and medical fields.

### 2.3. In Vitro Biological Activity and Biocompatibility of the Hydrogels

**Figure 4:**
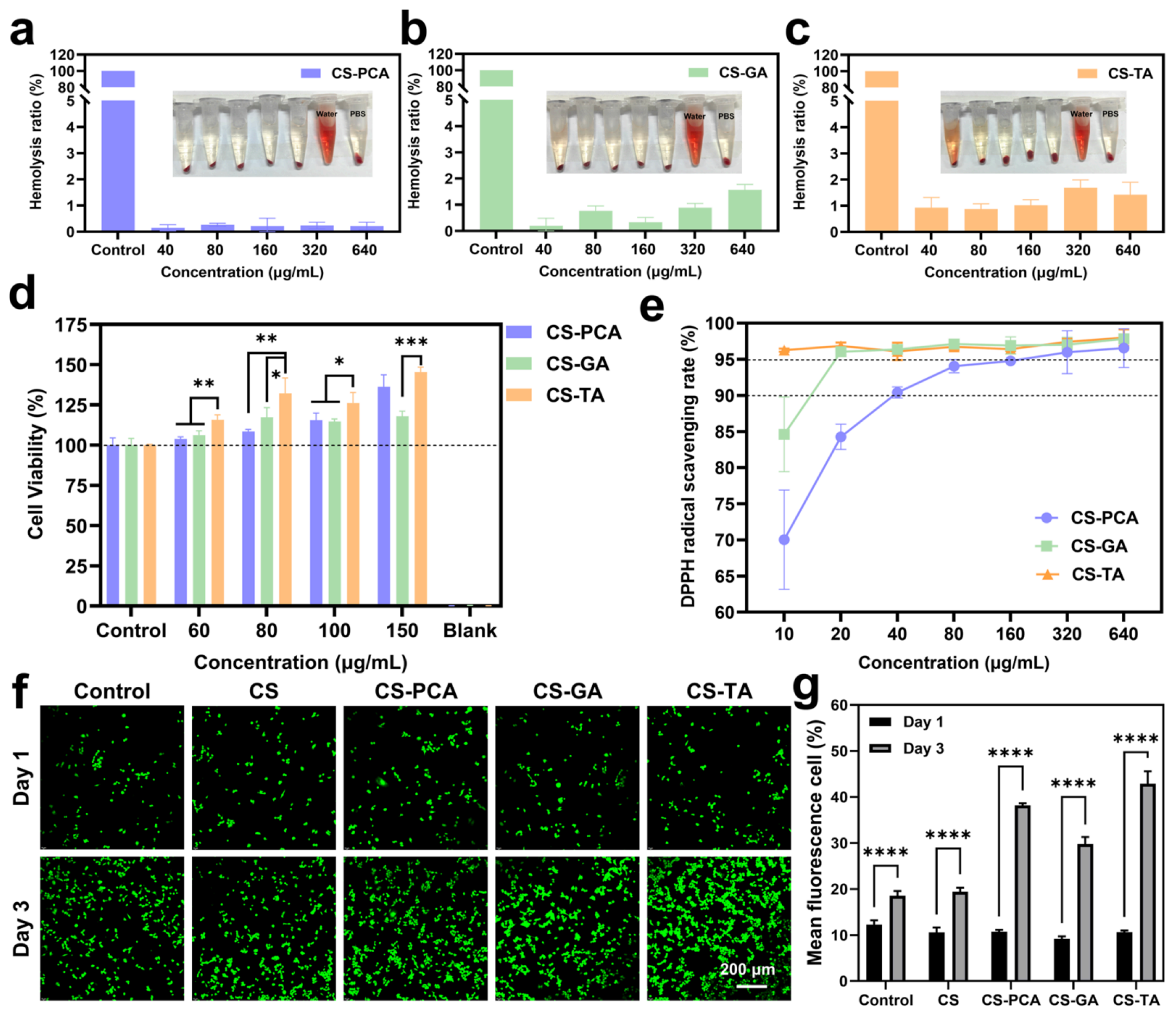
Biocompatibility of the hydrogels. Quantitative analysis of in vitro hemocompatibility and hemolysis rate of (**a**) CS-PCA; (**b**) CS-GA, and (**c**) CS-TA (*n* = 6). (**d**) L929 cell viabilities after 48 h of culture for CCK-8 assays (*n* = 6), and the dashed line represents the 100% level. (**e**) DPPH radical-scavenging rates of CS-PCA, CS-GA and CS-TA (*n* = 6), and the two dashed lines in panel (**b**) represent the 90% and 95% levels, respectively. (**f**) Representative live-cell fluorescence images of L929 cells after incubation with cell culture medium or hydrogel extract (150 μg/mL) for 1 and 3 days (scale bar = 200 μm). (**g**) Quantitative analysis of the percentage of live cells in panel (**f**) after incubation with hydrogel lixivium (150 μg/mL) for 1 and 3 days. Data are presented as mean ± SD (*n* = 3 independent experiments). Statistical analysis was performed using two-way ANOVA followed by Tukey’s multiple-comparison test. * *p* < 0.05, ** *p* < 0.01, *** *p* < 0.001, **** *p* < 0.0001.

The antioxidant activity of hydrogels enables effective neutralization of excess reactive oxygen species in the wound microenvironment, which is critical for alleviating oxidative stress, breaking the cycle of inflammation, and promoting normal wound healing progression. The DPPH radical-scavenging activities of the chitosan hydrogels (CS-PCA, CS-GA, and CS-TA) grafted with different phenolic acids are shown in Figure 4e. All the materials exhibited concentration-dependent antioxidant effects. CS-TA demonstrated the strongest scavenging capacity, reaching approximately 95% at 640 μg/mL, which is attributable to the high density of reactive phenolic hydroxyl groups in the tannic acid structure. CS-GA also showed potent activity, achieving approximately 90% scavenging, which is consistent with the known efficacy of gallic acid. In comparison, CS-PCA, which was grafted with protocatechuic acid, displayed more moderate antioxidant activity, with a maximum of 75%, reflecting its simpler molecular structure with fewer hydroxyl groups. The results confirm that the antioxidant performance of the hydrogels is directly influenced by the type and molecular structure of the incorporated phenolic acid.

Biocompatibility testing is a key indicator for determining whether bioactive materials can be used as wound dressings. An in vitro hemolysis assay was used to evaluate the hemocompatibility of the hydrogels. The supernatants in the CS-GA, CS-PCA, and CS-TA hydrogel groups were transparent and clear. The quantitative data also indicated that the hydrogel groups were below the 5% upper limit for hemolysis (Figure 4a–c).

Low cytotoxicity is essential for hydrogel wound dressings. Fibroblasts are essential for rebuilding the extracellular matrix during the wound-healing process [31]. Mouse embryonic fibroblasts (L929) were used as template cells to study the biocompatibility of the polyphenol–chitosan hydrogels. Cell compatibility was assessed using a CCK-8 cytotoxicity assay and live/dead cell staining. After L929 fibroblasts were cocultured with different concentrations of CS-PCA, CS-GA, and CS-TA for 48 h, all three materials exhibited good cell compatibility within the concentration range of 60–150 μg/mL, with cell survival rates all above 100% (Figure 4d). Notably, compared with the CS-PCA and CS-GA groups, the CS-TA group maintained extremely high cell survival rates even with increasing concentration, indicating a certain cell proliferation-promoting effect. These results highlight the unique advantage of CS-TA in cell proliferation, potentially related to the excellent antioxidant and cytoprotective properties of tannic acid [32]. Live/dead staining (Figure 4f) at 1 and 3 d revealed only a small number of dead cells (red fluorescence) in each group, with the vast majority being live cells (green fluorescence), further indicating that the hydrogels promoted cell proliferation, which was consistent with the results we obtained with the CCK-8 kit.

### 2.4. In Vitro Antibacterial Properties of Hydrogels

**Figure 5:**
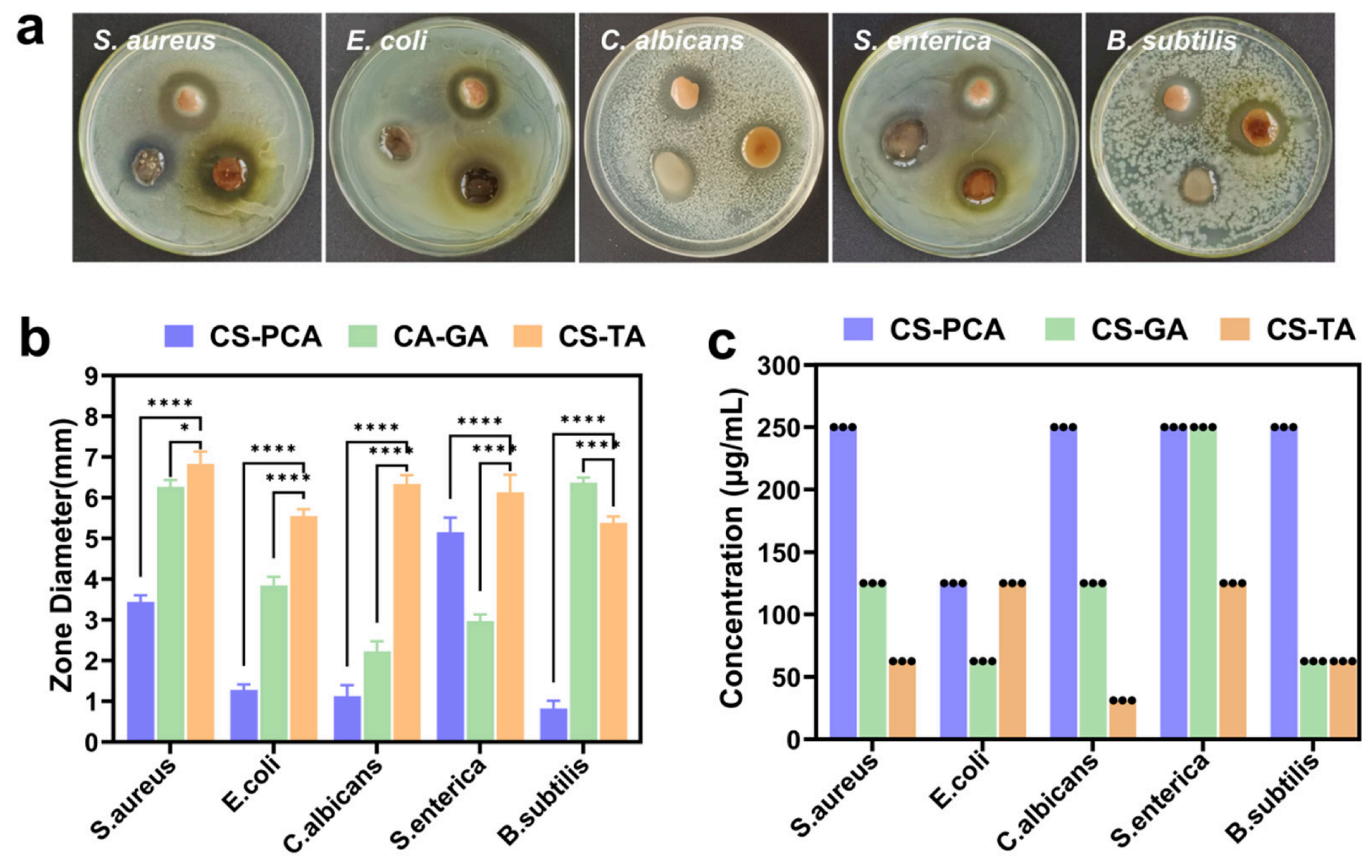
(**a**) Representative agar plates showing the antibacterial effects of CS-PCA, CS-GA, and CS-TA against *S. aureus*, *E. coli*, *C. albicans*, *S. enterica*, and *B. subtilis* (dilution: 10^4^). (**b**) Quantitative characterization of the bacteriostatic effect against *S. aureus*, *E. coli*, *C. albicans*, *S. enterica* and *B. subtilis* by using the inhibition zone method (*n* = 3 independent experiments). (**c**) The minimal inhibitory concentration (MIC) of the hydrogels against each strain was determined using the broth microdilution method (Dots indicate independent experimental replicates, *n* = 3). Statistical analysis was performed using one-way ANOVA followed by Tukey’s multiple-comparison test. * *p* < 0.05, **** *p* < 0.0001.

Hydrogel dressings with excellent antibacterial properties can create a wound barrier to inhibit bacterial infection effectively. To accurately quantify the test bacterial suspensions, standard curves of McFarland turbidity versus colony count were constructed for each bacterial species, which showed good linearity, with r^2^ values greater than 0.94 (Figure S6, Supporting Information). The antibacterial activities of the hydrogels against *Staphylococcus aureus* (*S. aureus*), *Escherichia coli* (*E. coli*), *Bacillus subtilis* (*B. subtilis*), *Salmonella enterica* (*S. enterica*), and *Candida albicans* (*C. albicans*) were evaluated using the agar diffusion method and MIC testing [33]. *S. aureus* and *C. albicans* are commonly associated with skin wounds. In our previous preliminary experiments, the CS hydrogel did not produce distinct inhibition zones against *S. aureus*, *E. coli*, or *C. albicans* (Figure S7, Supporting Information). In contrast, as shown in Figure 5a, the CS-GA, CS-PCA, and CS-TA hydrogels had antibacterial effects on five microorganisms. Moreover, for *S. aureus*, the calculated inhibition zones were 6.83 ± 0.30 (CS-TA), 6.27 ± 0.17 (CS-GA), and 3.44 ± 0.17 (CS-PCA) mm. For E. coli, the inhibition zones were 5.55 ± 0.17 (CS-TA), 3.84 ± 0.22 (CS-GA), and 1.28 ± 0.14 (CS-PCA) mm. For *C. albicans*, the inhibition zones were 6.34 ± 0.21 (CS-TA), 2.23 ± 0.24 (CS-GA), and 1.13 ± 0.27 (CS-PCA) mm. For *S. enterica*, the inhibition zones were 6.13 ± 0.44 (CS-TA), 2.97 ± 0.16 (CS-GA), and 5.15 ± 0.36 (CS-PCA) mm. For *B. subtilis*, the inhibition zones were 5.38 ± 0.16 (CS-TA), 6.37 ± 0.12 (CS-GA), and 0.83 ± 0.19 (CS-PCA) mm. The results (Figure 5b) indicated that the antibacterial activities of the different hydrogels varied significantly and exhibited unique selective inhibition spectra against different microorganisms. CS-TA demonstrated broad-spectrum and potent antibacterial effects against the most tested strains, particularly against *S. aureus*, *C. albicans*, and *S. enterica*. CS-GA showed the strongest inhibitory activity against *B. subtilis* and *S. aureus.* In contrast, compared with CS-GA and CS-TA, CS-PCA was most effective against *B. subtilis*. These differences clearly demonstrate that the type of phenolic acid (TA, GA, or PCA) grafted onto chitosan (CS) is a critical factor determining the synergistic antibacterial activity and selectivity.

As shown in Figure 5c, the MIC results demonstrated that CS-TA exhibited the most potent and broad-spectrum antibacterial activity, followed by CS-GA and CS-PCA. Tannic acid likely exerts its antibacterial effects by exacerbating oxidative stress [34], inhibiting efflux pumps [35], and disrupting cell wall synthesis [36], thereby synergistically compromising bacterial viability. CS-GA also displayed strong antibacterial activity, albeit slightly inferior to that of CS-TA. Notably, compared with Gram-negative bacteria, all materials were generally more effective against Gram-positive bacteria, possibly due to the protective outer membrane of the latter [37]. These findings confirm the significant advantage of polyphenol modification in enhancing the antibacterial properties of chitosan-based materials. These results demonstrate that the CS-GA, CS-PCA, and CS-TA hydrogels possess considerable antibacterial activity against skin infection-related pathogens such as *S. aureus*. The CS-TA hydrogel exhibited outstanding broad-spectrum efficacy against all five strains tested.

It should also be noted that the antibacterial evaluation in this study was mainly based on agar diffusion and broth microdilution MIC assays. These basic assays demonstrate the initial antibacterial activity of the hydrogels but do not fully reveal the antibacterial mechanism. Future studies should include time–kill curves, membrane-integrity assays, biofilm inhibition or eradication tests, bacterial morphology observation, and ROS-related analyses to further clarify the antibacterial pathways of the polyphenol–chitosan hydrogels.

### 2.5. In Vivo Wound Healing with Hydrogels

**Figure 6:**
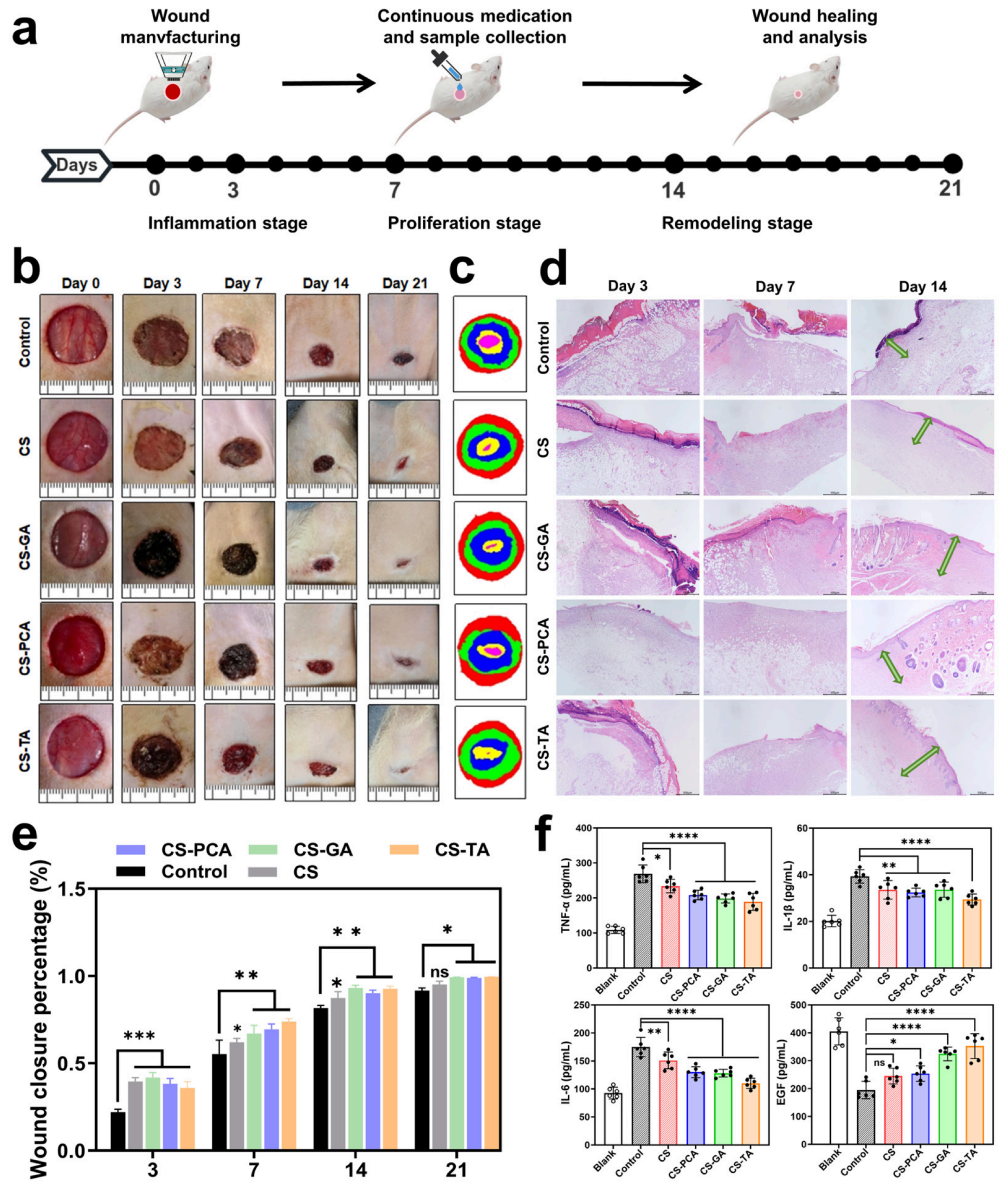
Wound healing assessment in vivo. (**a**) Schematic diagram of the timeline of the SD rat tests on the therapeutic effect of the wound dressing. (**b**) Representative images of wound closure at different time points in different treatment groups (scale bar = 10 mm, *n* = 6 independent experiments). (**c**) Schematic diagram of wound evolution in different groups (ImageJ 1.53k software). (**d**) H&E staining of wound tissues at various time points. The arrows indicate newly formed dermal tissue (scale bar = 500 μm). (**e**) Corresponding wound areas of the control, CS, CS-PCA, CS-GA, and CS-TA groups at 0, 3, 7, 14, and 21 d (*n* = 6 independent experiments). (**f**) TNF-α, IL-1β, IL-6 and EGF expression on Day 7 was determined by ELISA, The black and white dots in the figure represent individual data points (*n* = 6 independent experiments). All the data are presented as the mean ± SD. The significant differences in the corresponding data were analyzed using a two-tailed Student’s *t* test or one-way ANOVA. The statistically significant differences are expressed as * *p* < 0.05, ** *p* < 0.01, *** *p* < 0.001, **** *p* < 0.0001, and ns (not significant) compared with the control group.

The in vitro results demonstrated that polyphenol–chitosan hydrogels have stable mechanical properties and good tissue adhesion, antibacterial activity, biocompatibility, and cell proliferation-promoting activity with high biocompatibility with the skin; thus, we speculated that these hydrogels could effectively accelerate wound healing in the body. We therefore further evaluated the in vivo wound-healing properties of the hydrogels through a full-thickness wound model with a diameter of 20 mm established on the backs of the rats. The process of in vivo animal testing is shown in Figure 6a.

Figure 6b,c shows digital images and schematic diagrams of wound healing in the five treatment groups (physiological saline, CS, CS-PCA, CS-GA, and CS-TA) at 0, 3, 7, 14, and 21 d. The first treatment was administered immediately after wound induction. One hour post-application, slight bleeding and exudation were observed in the control and CS groups, with the CS hydrogel showing noticeable loss due to exudate washout. In contrast, the CS-GA, CS-PCA, and CS-TA hydrogels adhered firmly to the wound surface, effectively absorbed exudate, and formed a protective barrier, thereby reducing the risk of external infection, highlighting their excellent adhesion and swelling properties.

By Day 3, wound contraction and scab formation were observed in all the groups. The control group exhibited significant redness, swelling, and purulent discharge under the scab. Compared with the control group, the polyphenol hydrogel groups formed thicker scabs without signs of redness or swelling, indicating a better healing response. On Day 7, the wound areas in the CS-GA, CS-PCA, and CS-TA groups were significantly smaller than those in the control group, with partial scab detachment observed. On Day 14, the original scabs in the polyphenol hydrogel groups had completely detached, with new scab formation noted during the treatment period. Compared with that in the control group, wound healing in the experimental group was significantly improved and slightly superior to that in the CS group. On Day 21, the unhealed wounds (3–6 mm in diameter) remained in the control group, whereas the wounds in the polyphenol hydrogel groups were almost completely healed, with diameters generally less than 2 mm. Wound overlay images and statistical analysis of the healing rates consistently demonstrated that the CS-GA, CS-PCA, and CS-TA hydrogels significantly enhanced wound healing throughout the treatment period (Figure 6e).

To further assess the healing efficacy of the regenerated skin, hematoxylin and eosin (H&E) staining was performed on the wound tissues collected at 3, 7, and 14 d (Figure 6d). During the inflammatory and proliferative phases, the structural integrity of the wounds in all groups was incomplete, characterized by the absence of an epidermal layer, hair follicles, and blood vessels, along with a sparse and disorganized dermis. On Day 3, varying degrees of inflammatory responses were observed across the groups, marked by significant inflammatory cell infiltration and notable foreign body reactions. On Day 7, the hydrogel-treated groups showed the formation of a new epidermal layer, accompanied by substantial granulation tissue and newly formed blood vessels in the dermis. During the inflammatory phase of wound repair, parenchymal cells often struggle to accomplish complete tissue restoration. Granulation tissue plays a vital role in this process, promoting the dissolution and absorption of necrotic tissue and foreign material, filling tissue defects, and ultimately maturing into scar tissue, thereby facilitating wound closure [38]. Consequently, the thickness of granulation tissue serves as a key indicator for evaluating the effectiveness of wound healing. After 14 d of treatment, granulation tissue and blood vessels were visible in all groups, indicating that wound repair had entered the remodeling phase. Notably, the granulation tissue was thicker in the hydrogel groups than in the control group, demonstrating the ability of the hydrogels to promote granulation tissue proliferation and accelerate wound healing effectively.

Treatment with the CS-GA, CS-PCA, and CS-TA hydrogels significantly reduced the expression levels of TNF-α, IL-6, and IL-1β, which play important roles in the regulation of inflammation and trauma [39]. Among the three polyphenol–chitosan hydrogels, the CS-TA hydrogel demonstrated better inhibitory effects on IL-6 and IL-1β. Epidermal growth factor (EGF) is crucial for tissue regeneration and promotes skin cell proliferation, differentiation and metabolism. Impaired wound healing is often associated with downregulated EGF expression. ELISA results confirmed that the protein expression levels of EGF were markedly greater in the hydrogel-treated groups than in the control group (Figure 6f), demonstrating the ability of these polyphenol–chitosan hydrogels to promote EGF expression and facilitate skin repair.

## 3. Conclusions

In this study, CS-PCA, CS-GA, and CS-TA hydrogels were constructed within the same chitosan platform and compared under a unified framework in terms of structure, mechanics, swelling, remaining-weight behavior, adhesion, antibacterial activity, and wound-healing performance. All three hydrogels formed homogeneous porous networks and outperformed unmodified CS in viscoelasticity, wet adhesion, and bioactivity, but their advantages were clearly distinct. Among them, CS-TA showed the highest modulus, the strongest step-strain recovery, the most pronounced swelling under acidic conditions, and the best overall broad-spectrum antibacterial and pro-healing performance, indicating that TA is more favorable for building a function-dense and dynamically reversible hydrogel network. This advantage is likely related to the high density of phenolic hydroxyl groups and multivalent interactions provided by TA, which can generate a denser network through hydrogen bonding, aromatic interactions, and multipoint association with chitosan chains [40]. Its antibacterial superiority may also involve membrane-related perturbation, although this still requires direct validation by membrane damage, ROS, and biofilm assays [41,42].

By contrast, CS-GA showed stronger remaining-weight retention, suggesting better structural stability in aqueous media and a potential advantage when longer material residence or sustained coverage is needed. CS-PCA displayed a more balanced profile in microstructure, mechanical stability, swelling, and remaining-weight change, but its antibacterial activity was weaker than that of CS-GA and CS-TA, making it more suitable as a balanced hydrogel carrier rather than a strongly antibacterial one. Overall, CS-TA, CS-GA, and CS-PCA stand out, respectively, for higher functional density, stronger structural persistence, and more balanced overall performance, providing a practical basis for differentiated material selection according to wound type and healing stage.

Several limitations should be acknowledged. First, release, permeation, and diffusion studies were not included because the present work focused on comparing the structure–property–function relationships of three polyphenol–chitosan hydrogel matrices. Second, the stability evaluation was limited to short-term remaining-weight behavior in PBS, and accelerated or stress stability studies were not performed. Third, the in vivo sample size was determined according to the experimental endpoint design rather than by formal power analysis. Fourth, the antibacterial assessment was based on agar diffusion and MIC assays; therefore, additional mechanistic studies, including biofilm assays, membrane-damage analysis, and time-kill kinetics, are still needed. These issues will be addressed in future work to further support the pharmaceutical translation of this hydrogel system.

## 4. Materials and Methods

### 4.1. Materials

Chitosan (CS) (degree of deacetylation%: >90; Mw: 92.3 kDa (the detailed SEC-MALS characterization report in Figure S8, Supporting Information) was obtained from Shanghai Macklin Biochemical Co., Ltd. (Shanghai, China). Tannic acid (TA), protocatechuic acid (PCA) and gallic acid (GA) were obtained from Beijing InnoChem Science & Technology Co., Ltd. (Beijing, China).Absolute ethanol was purchased from Tianjin Jindong Tianzheng Fine Chemical Reagent Factory. (Tianjin, China).Acetic acid glacial was obtained from Tianjin Fengchuan Chemical Reagent Co., Ltd. (Tianjin, China). (3-Dimethylaminopropyl)-3-ethylcarbodiimide hydrochloride (EDC) and N-hydroxysuccinimide (NHS) were obtained from Shandong Ke Yuan Biochemical Co., Ltd. (Jinan, China). L-929 (Cat No. FH0534) was provided by Shanghai Fuheng Biotechnology Co., Ltd. (Shanghai, China). Luria–Bertani (LB) was purchased from Beijing Solarbio Biotechnology Co., Ltd. (Beijing, China). Nutrient agar medium was supplied by Guangdong HuanKai Microbial Technology Co., Ltd. (Guangzhou, China) *Staphylococcus aureus* (*S. aureus*), *Escherichia coli* (*E. coli*), *Candida albicans* (*C. albicans*), *Salmonella enterica* (*S. enterica*), and *Bacillus subtilis* (*B. subtilis*) were obtained from BeNa Culture Collection (BNCC). *Escherichia coli* EM was from Shanghai Wokai Biological Technology Co., Ltd. (Shanghai, China). Paraformaldehyde was supplied by Sangon Biotech (Shanghai) Co., Ltd. (Shanghai, China). Mouse TNF-α, IL-1β, IL-6 and EGF ELISA kits were purchased from Shanghai MLBIO Biotechnology Co., Ltd. (Shanghai, China). Hematoxylin–Eosin (H&E) staining reagents were from Beijing Solarbio Science & Technology Co., Ltd. (Beijing, China).

### 4.2. Synthesis of Hydrogels

A 40 mg/mL chitosan (CS) solution was prepared in 1% (*v*/*v*) acetic acid. NHS and EDC solutions were prepared at 230 mg/mL and 384 mg/mL, respectively, and PCA, GA, and TA solutions were each prepared at 10 mg/mL. Before the final formulations were determined, extensive single-factor preliminary experiments were first carried out to identify a feasible composition range for each polyphenol system. Based on these preliminary results, a secondary screening was then performed by fixing the CS solution volume at 40 mL and varying the volume of the polyphenol solution to 2, 4, 6, 8, and 10 mL. The amounts of EDC and NHS were set according to the molar amount of chitosan repeat units. The gelation behavior of the resulting formulations was evaluated by macroscopic observation and vial-inversion testing (Figure S1 Supporting Information). The formulation containing 8 mL polyphenol solution showed a more suitable overall gel state and was therefore selected for the subsequent study. Accordingly, 40 mL of CS solution, 5 mL of EDC solution, 5 mL of NHS solution, and 8 mL of PCA, GA, or TA solution were mixed under stirring at 55 °C and 1500 rpm to obtain CS-PCA, CS-GA, and CS-TA hydrogels.

### 4.3. Structure Analysis

The prepared composite hydrogels were dialyzed for 48 h to remove organic solvents and unreacted impurities. The samples were then freeze-dried at −60 °C for 72 h to obtain a purified lyophilized powder.

The lyophilized hydrogel samples were dissolved in deionized water and analyzed using a UV–Vis spectrophotometer within the wavelength range of 190–500 nm. Absorption spectra of CS-GA, CS-PCA, and CS-TA were recorded.

Lyophilized hydrogel samples were also analyzed by ^1^H NMR. Spectra were recorded at 25 °C on a 600 MHz nuclear magnetic resonance spectrometer. The modified hydrogel samples were dissolved in 2% CD_3_COOD/D_2_O, and the spectra were used to compare the characteristic aromatic proton signals of the polyphenols with those of the chitosan backbone.

A small amount of each dried sample (CS-GA, CS-PCA, and CS-TA) was subjected to Fourier transform infrared (FT-IR) spectroscopy. Spectra were collected from 4000 to 400 cm^−1^ to identify characteristic functional groups.

The freeze-dried samples were sputter-coated with gold and observed using a benchtop scanning electron microscope (ZEM18, Shanghai Ailang Instrument Co., Ltd., Shanghai, China). SEM images of the three hydrogels were obtained to examine their morphologies.

### 4.4. Rheological Properties

The rheological properties of the hydrogels (CS, CS-PCA, CS-GA, and CS-TA) were characterized using a hybrid rheometer (MCR52, Anton Paar). Measurements were conducted at a fixed strain of 1% with an angular frequency sweep from 0.1 to 100 rad/s to determine the storage modulus (G′) and loss modulus (G″).

### 4.5. Swelling Ratio and Degradation Rate

To evaluate the pH-dependent swelling behavior, 2 mL of each hydrogel (CS, CS-PCA, CS-GA, and CS-TA) was aspirated using a disposable syringe and transferred into PBS (pH = 5.0, pH = 7.2). At 2 h intervals, samples were removed, surface moisture was blotted away, and their weights were recorded. The swelling ratio was calculated as follows:

Swelling ratio (%) = [(*m_t_* − *m*_0_)/*m*_0_] × 100%

where *m*_0_ is the initial mass of the freshly prepared hydrogel and *m_t_* denotes the mass of the hydrogel at a given time point after water absorption.

To evaluate the in vitro degradation behavior, freshly prepared hydrogels (CS-PCA, CS-GA, and CS-TA) of equal mass were placed into pre-weighed clean, dry glass vials. The initial total weight was recorded, after which 10 mL of phosphate-buffered saline (PBS) at different pH values (pH = 5.0 and pH = 7.2) was added to each vial, and the vials were incubated in a 37 °C water bath. At predetermined time points (0 h, 6 h, 12 h, 1 d, 2 d, 3 d, 4 d, 5 d, 6 d, and 7 d), the samples were removed, the liquid inside the vial and on the surface was gently blotted with filter paper, and the weight was recorded. An equal volume of fresh PBS was then replenished. The remaining weight percentage was calculated using the following formula:

Remaining weight (%) = (*W_t_* − *W*_0_)/(*W_s_* − *W*_0_) × 100%

where *W*_0_ is the weight of the empty glass vial, *W_s_* is the initial total weight of the vial with the hydrogel, and *W_t_* is the weight of the vial with the remaining hydrogel at a given degradation time point.

### 4.6. Self-Healing and Injectability

For the self-healing evaluation, a hydrogel sample was cut into two pieces. The cut surfaces were placed in contact and observed for autonomous fusion without external intervention. Injectability was assessed by loading a hydrogel sample onto a disposable syringe and extruding it onto a flat surface to examine the continuity and shape fidelity of the extruded strand.

### 4.7. Adhesive Properties

The adhesive properties were tested by applying the hydrogel to various moist biological tissues, including bone, muscle, lung, stomach, spleen, kidney, heart, blood vessels, and liver, as well as to various other organic and inorganic substrates.

### 4.8. Assessment of Cell Viability Using a CCK-8 Assay

Cell viability was evaluated using a CCK-8 assay following treatment with the hydrogel extracts. L929 cells were seeded into 96-well plates at a density of 5 × 10^3^ cells per well and cultured in DMEM for 24 h. Hydrogel samples (CS-PCA, CS-GA, and CS-TA) at various concentrations (60, 80, 100, 150 µg/mL) were sterilized under UV light and subsequently immersed in complete DMEM for 24 h to prepare the extraction media. After the initial cell adhesion period, the culture medium was replaced with the respective hydrogel extracts, and the cells were incubated for another 24 h. Following incubation, 10% (*v*/*v*) CCK-8 reagent was added to each well, and the plates were incubated in the dark for 2 h. The optical density (OD) at 450 nm was measured using a microplate reader (Tecan, Beijing, China). Cell viability was calculated as follows:

Cell viability (%) = [(*OD_experimental_* − *OD_blank_*)/(*OD_control_* − *OD_blank_*)] × 100%

where *OD_experimental_* refers to the absorbance of the experimental wells (including cell, medium, CCK-8 solution and hydrogel extract), *OD_control_* refers to the absorbance of the negative control wells (cells, medium, and CCK-8 solution without hydrogel extract), and *OD_blank_* corresponds to the absorbance of the background control wells (medium and CCK-8 solution only without cells or hydrogel extract).

### 4.9. Calcein-AM/PI Staining

Cell viability was further evaluated using calcein-AM/PI staining. L-929 cells were seeded into 20 mm confocal laser culture dishes and incubated for 12 h to allow complete adhesion. The culture medium was then aspirated, and the cells were washed three times with PBS. After the respective treatments, the cells were washed again three times with PBS. The cells were subsequently incubated with calcein-AM/PI staining solution in the dark for 20 min. Following incubation, the staining solution was removed, and the cells were washed three times with PBS. Fluorescence images were acquired using a confocal laser scanning microscope (Leica, Wetzlar, Germany).

### 4.10. Hemolytic Activity Assay

Hemolysis testing was performed using blood samples obtained from healthy Sprague–Dawley (SD) rats via retro-orbital bleeding. The blood samples were centrifuged at 3000 rpm for 5 min, after which the supernatant was removed. The resulting red blood cells (RBCs) were repeatedly washed with PBS and centrifuged at 5000 rpm until the supernatant became clear. The pelleted RBCs were then dispersed in PBS to prepare a 2% (*v*/*v*) RBC suspension. The following experimental groups were established: a positive control (ultrapure water), a negative control (PBS), and PBS containing serial concentrations (40, 80, 160, 320, 640 µg/mL) of the respective hydrogels (CS-PCA, CS-GA, and CS-TA). For each sample, 800 µL of the test solution was mixed with 200 µL of the RBC suspension to form a 1.0 mL homogeneous mixture. The mixtures were incubated at 37 °C in a constant-temperature shaker for 2 h. After incubation, the samples were centrifuged at 5000 rpm for 5 min. The supernatant from each sample was transferred to a 96-well plate, and its optical density (OD) was measured at 570 nm using a microplate reader. The hemolysis ratio was calculated using the following formula:

Hemolysis ratio (%) = [(*OD_sample_* − *OD_negative_*)/(*OD_positive_* − *OD_negative_*)] × 100%

where *OD_negative_*, *OD_positive_*, and *OD_sample_* represent the absorbance values of the negative control (PBS), positive control (ultrapure water), and test samples, respectively.

### 4.11. In Vitro Antimicrobial Experiment

The antimicrobial activities of the hydrogels (CS-PCA, CS-GA, and CS-TA) were evaluated against five common microbial strains: Gram-positive bacteria (*Staphylococcus aureus* and *Bacillus subtilis*), Gram-negative bacteria (*Escherichia coli* and *Salmonella enterica*), and a fungus (*Candida albicans*).

Frozen microbial suspensions were thawed, resuscitated, and cultured in LB liquid medium to the optimal conditions for use as the initial suspension in subsequent experiments. To accurately quantify the bacterial concentration, McFarland–Colony Forming Unit (MCF-CFU) standard curves were constructed for the five strains, providing a quantitative basis for determining the volume of the microbial suspension used in subsequent experiments.

The agar diffusion method was used to determine the inhibition zone width of each hydrogel group (CS-PCA, CS-GA, and CS-TA). *Candida albicans* was cultured in LB medium at 28 °C for 12 h, and the other strains were cultured at 37 °C to obtain microbial suspensions. The suspensions were then diluted 10^6^-fold, and 0.25 mL of the diluted suspension was inoculated and spread evenly onto the surface of nutrient agar plates. Subsequently, 0.2 mL of each hydrogel sample was drawn using a 1.0 mL disposable syringe and injected onto the inoculated plates. The plates were incubated in a constant-temperature incubator (*C. albicans* at 28 °C, other strains at 37 °C) for 24 h. After incubation, bacterial growth around the hydrogels was observed, and the width of the inhibition zone was measured. Each group was tested in triplicate.

To further assess antimicrobial activity, the minimum inhibitory concentration (MIC) of the hydrogels against each strain was determined using the broth microdilution method. Lyophilized hydrogel samples were dissolved in sterile LB liquid medium at a concentration of 1024 μg/mL. Twofold serial dilutions were performed using twelve sterile EP tubes per sample. Each tube initially contained 2 mL of LB medium. Two milliliters of the sample solution was added to the first tube. After mixing, 2.0 mL of the mixture was transferred to the second tube. This dilution process was repeated sequentially through the twelfth tube. Finally, 2.0 mL was removed from the twelfth tube and placed into an empty sterile EP tube as a blank control. Afterward, 0.2 mL of a microbial suspension (10^6^ CFU/mL) was added to each tube, except for the blank control. The tubes were mixed thoroughly and incubated at 37 °C for 48 h. The results were observed initially, and the McFarland turbidity was measured using a turbidimeter. The experimental groups included a negative control (containing the drug solution, no microbial suspension), a positive control (no drug solution, containing the microbial suspension), and a blank control (no drug solution, no microbial suspension).

### 4.12. In Vivo Full-Thickness Skin Wound-Healing Evaluation

Male Sprague–Dawley rats (200 ± 20 g, *n* = 72) were used. The sample size was determined based on the experimental endpoints, tissue-collection schedule, animal-use reduction, and common practice in preliminary full-thickness wound-healing studies. After a 7-day acclimatization period, the animals were randomly divided into six groups (*n* = 12 per group): blank, model, CS, CS-PCA, CS-GA, and CS-TA. The blank group received no wound creation or treatment. The model group received wound creation without treatment. The CS, CS-PCA, CS-GA, and CS-TA groups received the corresponding treatments. Group allocation was blinded during the in vivo experiment. Visually observed outcomes were independently evaluated by three investigators. However, no formal power analysis was performed before the in vivo experiment, which is a limitation of this study. The animal experiment was approved by the Medical Ethics Committee of Inner Mongolia Medical University (Approval No. YKD202504185). After intraperitoneal anesthesia with 20% urethane, a circular full-thickness skin wound (20 mm in diameter) was created on the back of each rat using a sterile biopsy punch. Treatments were applied once daily for 21 consecutive days. In each group, 3 rats were collected on days 3, 7, and 14 for histological analysis, and the remaining 3 rats were used for terminal wound evaluation and wound-area analysis. The wound sites were photographed on days 0, 3, 7, 14, and 21.

The wound areas were measured from the images using ImageJ software, and the wound healing rate was calculated as follows:

Closure rate (%) = [(*S*_0_ − *Sₜ*)/*S*_0_] × 100%

where *S*_0_ represents the original wound area and *Sₜ* represents the wound area on the recorded day.

### 4.13. Wound Tissue Histopathological Study

Wound tissues harvested at different time points (3, 7, and 14 days) were subjected to hematoxylin and eosin (H&E) staining. The tissue samples were fixed in 4% paraformaldehyde for 24 h, embedded in paraffin, and cut into 5 μm thick sections. The sections were then stained with H&E, and stained sections were imaged using an optical microscope. Collagen density and the number of new blood vessels in the histological skin samples were assessed using ImageJ software. For the animal experiment, a minimum of three biological replicates per group were performed, and H&E staining was conducted separately for each replicate.

### 4.14. In Vitro Inflammatory Cytokine Expression

Serum samples collected from the SD rats were stored at −80 °C. Prior to testing, the serum was thawed at 4 °C and allowed to equilibrate at room temperature for 5 min. The expression levels of TNF-α, IL-1β, IL-6, and EGF in the rat serum were measured using the appropriate ELISA kits according to the manufacturers’ instructions.

### 4.15. Statistical Analysis

Experimental data were processed using SPSS 26.0 statistical software and are presented as the mean ± standard deviation (SD). Statistical significance was determined by one-way analysis of variance (ANOVA), and significant effects were determined by two-tailed paired Student’s *t* test. Values of *p* < 0.05 were considered to indicate statistical significance. Graphs were generated using GraphPad Prism 10.3.0 software and OriginPro 2025 software. All experiments were performed with a minimum of three samples per group and repeated at least three times.

## Data Availability

The original contributions presented in this study are included in the article/Supplementary Material. Further inquiries can be directed to the corresponding authors.

## Supplementary Materials

The following supporting information can be downloaded at: https://www.mdpi.com/article/10.3390/gels12050448/s1. Figure S1—Preliminary formulation screening of CS-PCA, CS-GA, and CS-TA hydrogels. Figure S2—UV-Vis spectra of the hydrogels: (a) CS-PCA; (b) CS-GA; (c) CS-TA. Figure S3—^1^H NMR spectra of CS (2% CD_3_COOD/D_2_O, 600 MHz), PCA (D_2_O, 600 MHz), GA (D_2_O, 600 MHz) and TA (D_2_O, 600 MHz). Figure S4—SEM image of the lyophilized CS hydrogel (scale bar = 100 μm / 20 μm). Figure S5—Self-healing process of hydrogels: (a) CS-PCA; (b) CS-GA; (c) CS-TA. Figure S6—Standard curves of McF-CFU for different bacterial species. Figure S7—Agar plates treated with the CS hydrogel. Dilution times: 10^5^. Figure S8a—SEC-MALS chromatogram profiles of the chitosan sample. Figure S8b—Molar mass distribution of chitosan plotted against elution time.

## Abbreviations

CS	Chitosan
PCA	Protocatechuic acid
GA	Gallic acid
TA	Tannic acid
EDC	1-Ethyl-3-(3-dimethylaminopropyl) carbodiimide hydrochloride
NHS	N-hydroxysuccinimide
FT-IR	Fourier transform infrared
UV–Vis	Ultraviolet–Visible
*G*′	storage modulus
*G*″	loss modulus
*S. aureus*	Staphylococcus aureus
*E. coli*	Escherichia coli
*B. subtilis*	Bacillus subtilis
*S. enterica*	Salmonella enterica
*C. albicans*	Candida albicans
H&E	hematoxylin and eosin
