# Supplementary material for: Natural-Origin Bioadhesive Injectable Hydrogels Composed of Polyphenol and Chitosan with Antibacterial Activity for Wound Healing
Source: Gels. 2026 May 20;12(5):448. doi: 10.3390/gels12050448 (PMC13205482; doi:10.3390/gels12050448)
Supplement: Supplementary file 1 [file gels-12-00448-s001.zip › gels-4228733-supplementary.pdf]

Supporting information

Natural-Origin Bioadhesive Injectable Hydrogels Composed of Polyphenol and Chitosan with Antibacterial Activity for Wound Healing

Hongyu Zheng, Shikui Wu, Yujie Liu, Yuzhu Zhang, Yushu Xing, Jianye Wang, Xin Yue, Lijun Sun, Xiao Li, Ying Zhang, Jiannan Ma, Xiaoli Du, Yan Xue, Juan Yu, Huiwen Zhang * and Huanyun Wang *

School of Pharmacy, Inner Mongolia Medical University, Hohhot 010110, China; zhenghongyuzz@163.com (H.Z.); wushikui@immu.edu.cn (S.W.); 2025110105@stu.immu.edu.cn (Y.L.); zhangyuzhu5114@gmail.com (Y.Z.); fdas.a@163.com (Y.X.); nmgwangjianye@163.com (J.W.); yinlucky@immu.edu.cn (X.Y.); iamsunlijun1984@126.com (L.S.); lx_leexiao@163.com (X.L.); 20070176@immu.edu.cn (Y.Z.); jiannanma@163.com (J.M.); 20120018@immu.edu.cn (X.D.); xyxy2172@163.com (Y.X.); yujuan4893@163.com (J.Y.)

* Correspondence: 20180157@immu.edu.cn (H.Z.); whuanyun999@163.com (H.W.)

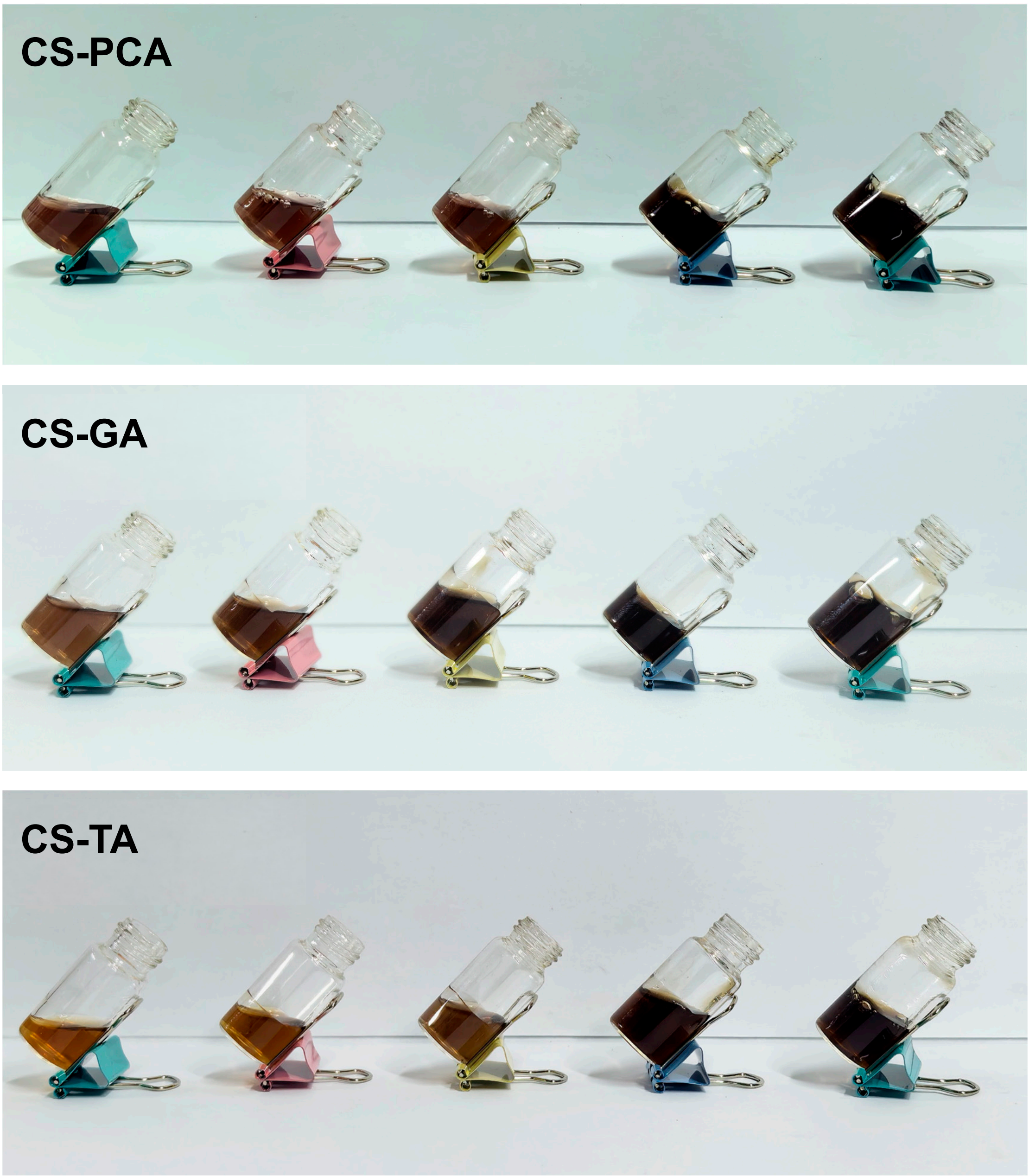

Figure S1. Preliminary formulation screening of CS-PCA, CS-GA, and CS-TA hydrogels. For each system, the volume of the polyphenol solution was set at 2, 4, 6, 8, and 10 mL from left to right, while the chitosan solution volume was fixed at 40 mL. The glass vials contain 2 mL aliquots of the prepared hydrogels for macroscopic observation.

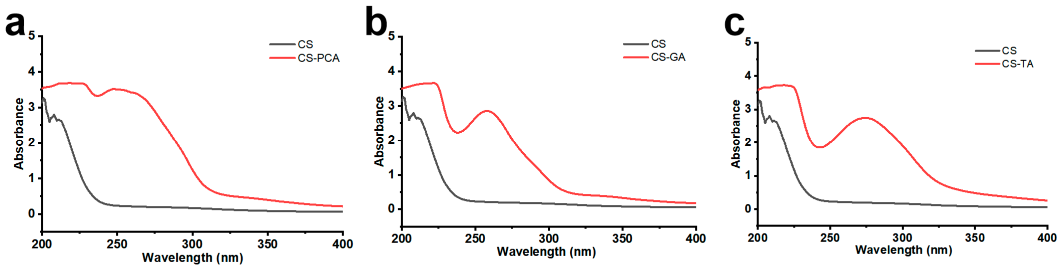

Figure S2. UV-Vis spectra of the hydrogels: a) CS-PCA; b) CS-GA; c)CS-TA.

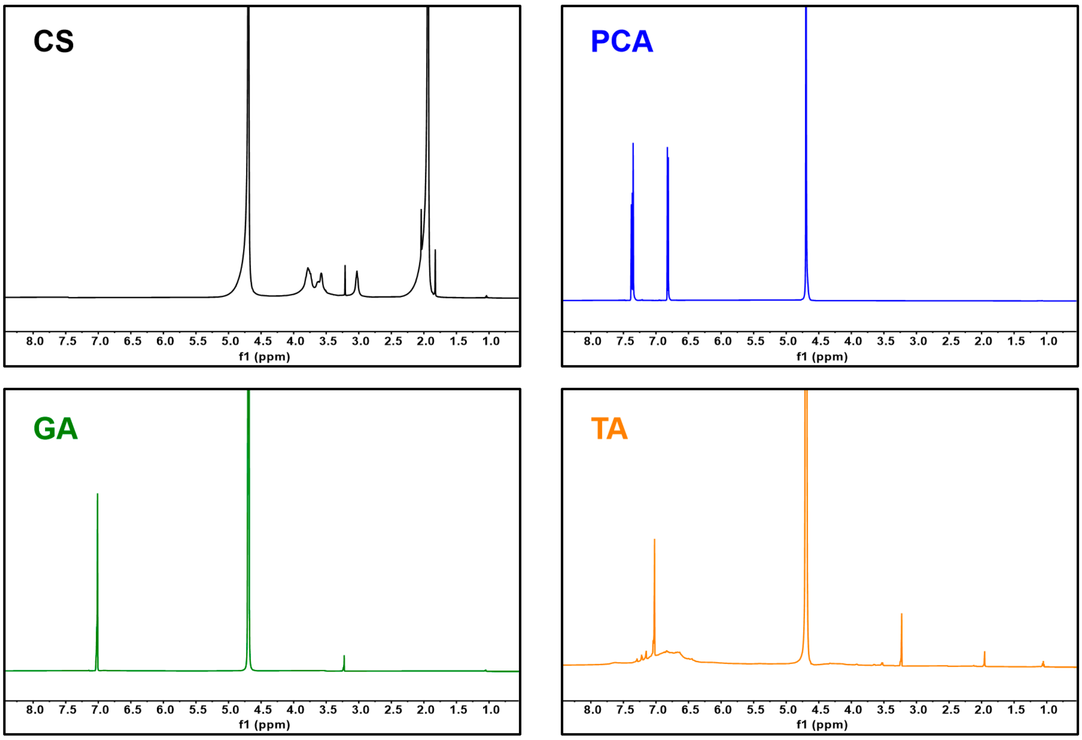

Figure S3. ^{1}H NMR spectra of CS (2% CD_3COOD/D_2O, 600 MHz), PCA (D_2O, 600 MHz), GA (D_2O, 600 MHz) and TA (D_2O, 600 MHz).

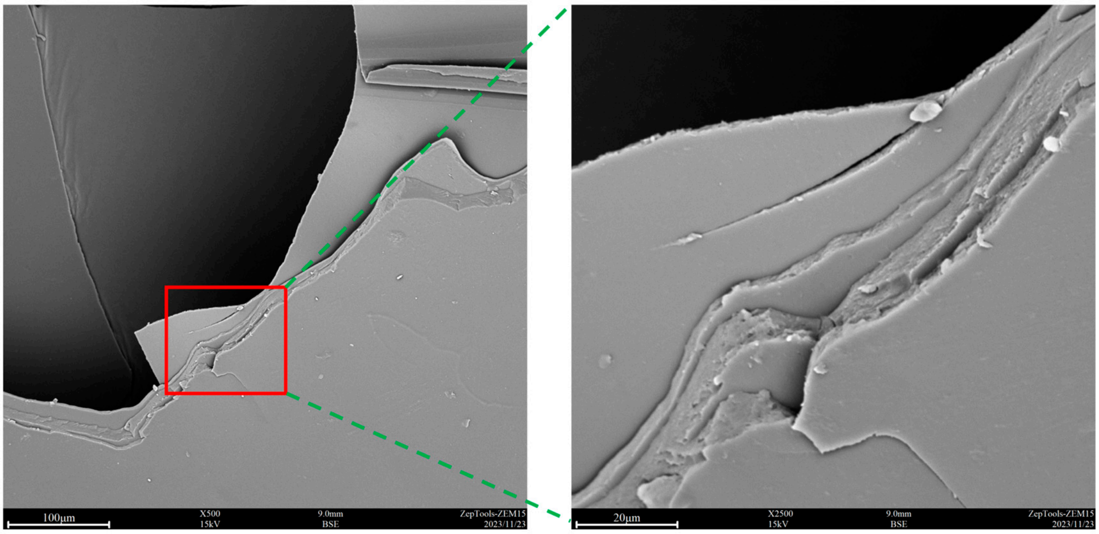

Figure S4. SEM image of the lyophilized CS hydrogel (scale bar = 100 μm / 20 μm).

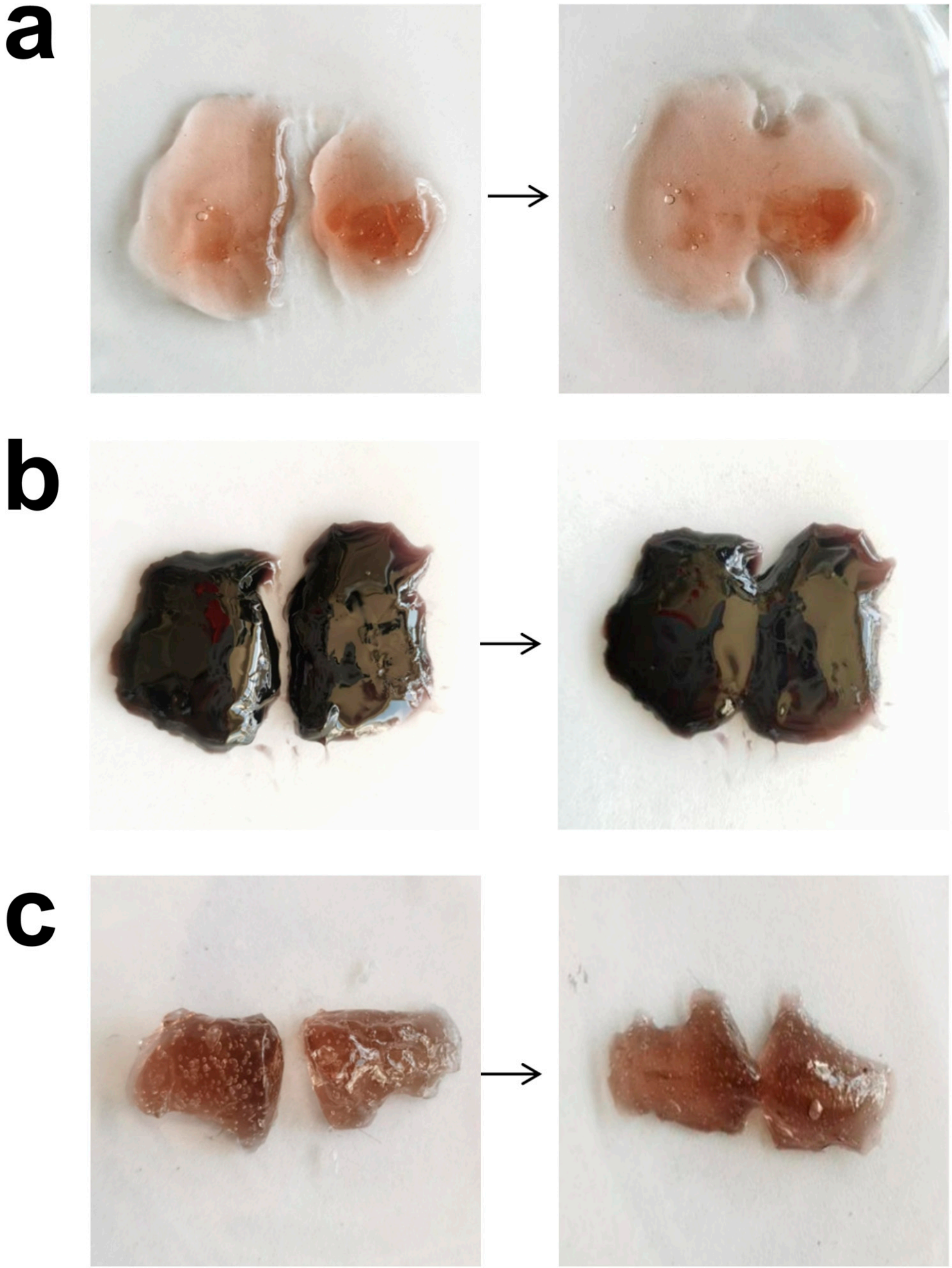

Figure S5. Self-healing process of hydrogels: a) CS-PCA; b) CS-GA; c) CS-TA.

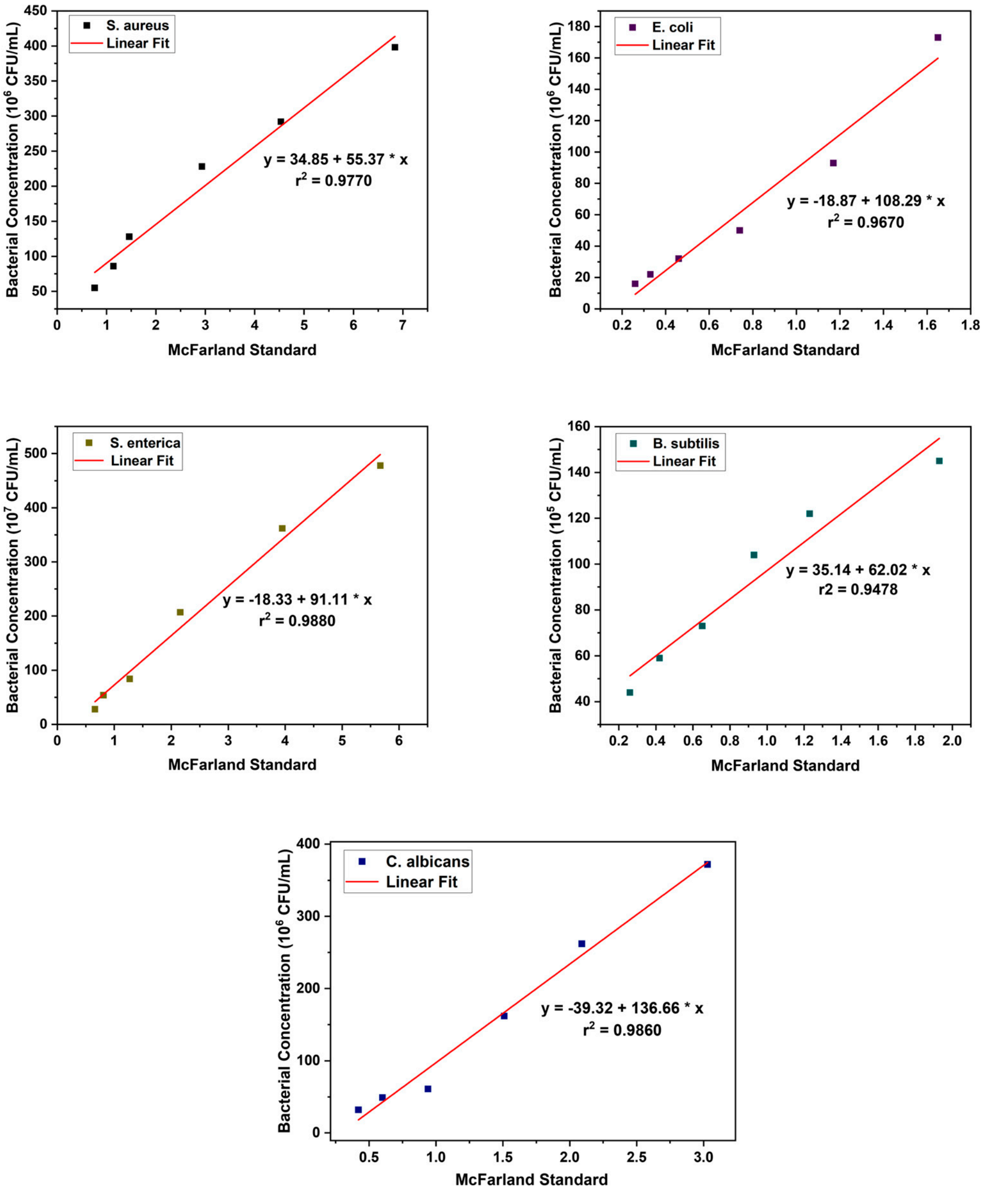

Figure S6. Standard curves of McF-CFU for different bacterial species.

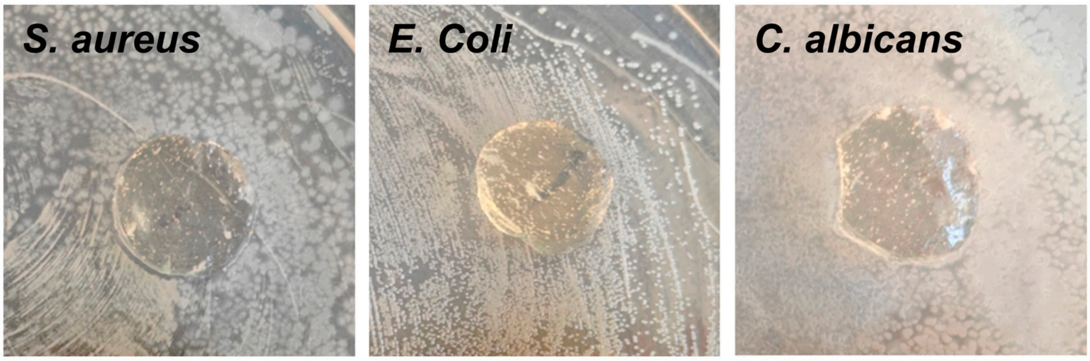

Figure S7. Agar plates treated with the CS hydrogel. Dilution times: 10^5.

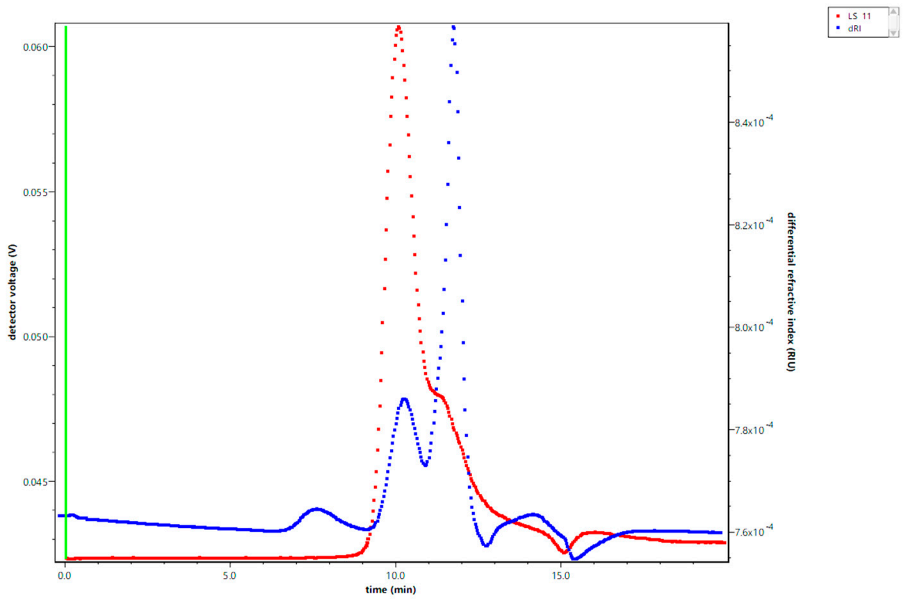

Figure S8a. SEC-MALS chromatogram profiles of the chitosan sample. The red trace corresponds to the light scattering (LS) detector signal, and the blue trace corresponds to the differential refractive index (dRI) detector signal.

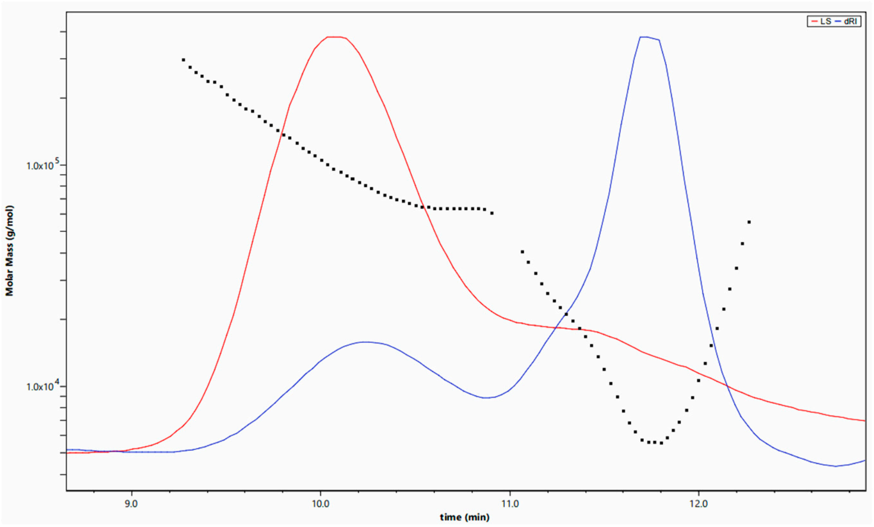

Figure S8b. Molar mass distribution of chitosan plotted against elution time. The red logarithmic scale points represent the absolute molar mass (Mw) calculated across the chromatographic peaks.
